# Understanding the form and function in Chinese bound foot from last-generation cases

**DOI:** 10.3389/fphys.2023.1217276

**Published:** 2023-09-19

**Authors:** Qichang Mei, Yaodong Gu, Julie Kim, Liangliang Xiang, Vickie Shim, Justin Fernandez

**Affiliations:** ^1^ Faculty of Sports Science, Ningbo University, Ningbo, China; ^2^ Research Academy of Grand Health, Ningbo University, Ningbo, China; ^3^ Auckland Bioengineering Institute, The University of Auckland, Auckland, New Zealand; ^4^ Department of Engineering Science, The University of Auckland, Auckland, New Zealand

**Keywords:** gait biomechanics, bone shape, functional adaptation, remodelling, bound foot

## Abstract

**Purpose:** Foot adaptation in the typically developed foot is well explored. In this study, we aimed to explore the form and function of an atypical foot, the Chinese bound foot, which had a history of over a thousand years but is not practised anymore.

**Methods:** We evaluated the foot shape and posture via a statistical shape modelling analysis, gait plantar loading distribution via gait analysis, and bone density adaptation via implementing finite element simulation and bone remodelling prediction.

**Results:** The atypical foot with binding practice led to increased foot arch and vertically oriented calcaneus with larger size at the articulation, apart from smaller metatarsals compared with a typically developed foot. This shape change causes the tibia, which typically acts as a load transfer beam and shock absorber, to extend its function all the way through the talus to the calcaneus. This is evident in the bound foot by i) the reduced center of pressure trajectory in the medial–lateral direction, suggesting a reduced supination–pronation; ii) the increased density and stress in the talus–calcaneus articulation; and iii) the increased bone growth in the bound foot at articulation joints in the tibia, talus, and calcaneus.

**Conclusion:** Knowledge from the last-generation bound foot cases may provide insights into the understanding of bone resorption and adaptation in response to different loading profiles.

## Introduction

The Chinese bound foot, which is not practised anymore, is a good example of an atypical foot form that demonstrates the form and function. The human foot has evolved and adapted in its shape and function due to remodelling to adapt to the environment (Ambrosi et al., 2019). The Chinese bound foot, also known as ‘foot binding,’ has its origins approximately between the late Tang dynasty (AD 618–907) and early Song dynasty (AD 960–1127) (Greenhalgh, 1977). This traditional custom has been practiced for over one thousand years, and it was banned in the early 20th century (Greenhalgh, 1977; Jackson, 1990). The binding practice was performed in early childhood for girls at 5–7 years of age, by bending the toes (except the large toe) underneath the plantar surface and wrapping the metatarsals towards the calcaneus using bandages, which would be replaced throughout life, thus forming a significantly shortened foot and prominently high arch with realigned bony structures (Stone, 2012). Although this practice was banned in the early 20th century, elderly females who lived in rural areas still continued to have their feet bound for some time after it was banned (Reischl et al., 2008; Richardson, 2009; Gu et al., 2013; Pan et al., 2013; Qin et al., 2015). The full-bound (FB) foot was defined as practicing foot binding throughout life, forming deformed toes and a high arch. The half-bound (HB) foot was defined as practicing foot binding and releasing the binding since the banning of the custom, also forming a deformed foot structure, particularly in the toes.

The foot posture differences between the FB, HB, and normal foot (NF) have been associated with reported differences in foot bone density. The shape characteristics of bound feet included an atypical high arch in the midfoot, vertically oriented calcaneus, dislocated phalanges, and lower bone density as observed in X-ray and/or computed tomography (CT) imaging studies (Munk and Poon, 1996; Richardson, 2009; Gu et al., 2013). However, the classification of the foot binding type was not reported, as the HB could only be found since the banning of this practice. While considering the binding practice (Greenhalgh, 1977; Reznikov et al., 2017), the toes (apart from the hallux) were flexed or curled underneath the plantar surface, which was believed to dislocate the phalanges from the metatarsals, thus losing the supporting function in the foot (Lambrinudi, 1932). The binding force folded the metatarsals towards the calcaneus, leading to a vertically aligned calcaneus as an extension of the shank and talus. Foot binding led to reduced physical activity and increased susceptibility to osteoporosis, but they did not have higher rates of fractures than typical feet likely due to compensation in activities and body balance (Qin et al., 2015).

Gait characteristics of females with bound feet have been reported including daily activities, such as walking (Cummings et al., 1997; Richardson, 2009; Lee, 2019). Gait experiments indicated an increased cadence, decreased stride length, and decreased range of motion at the knee and ankle joints in bound foot females (Zhang et al., 2015). This was possibly related to the deformed forefoot (metatarsals), leading to restricted ankle rollover during stance and reduced range of motion. Studies of walking footprint and plantar pressure distribution revealed focalised pressure at the heel region (Reischl et al., 2008; Gu et al., 2015) in the bound foot. From a functional perspective, the vertically oriented calcaneus was described as an extension of the lower leg for shock dampening (Reznikov et al., 2017), thus leading to the remodelling of the calcaneus (Reznikov et al., 2017). Evidence of bound foot gait adapting to this foot condition has been reported (Qin et al., 2015). Additional information including anthropometric, lifestyle questionnaires, calcaneus quantitative ultrasound, and high-resolution peripheral quantitative CT (HR-pQCT) reported higher risks of osteoporosis in the calcaneus than healthy females (Qin et al., 2015). In addition, the foot binding condition increased the risk of falling (Richardson, 2009).

The objective of this study is to examine two versions of the Chinese bound foot, the FB and HB, and compare them with a similarly aged typical female foot to demonstrate the form and function of the human foot. To our knowledge, this is the first time gait, plantar pressure, bone shape analysis, and finite element modelling have been integrated to evaluate the Chinese bound foot. The aim is to evaluate if foot plantar loading and foot posture can explain the adaptation in the bone shape and bone density.

## Materials and methods

### Participants

**FIGURE 1:**
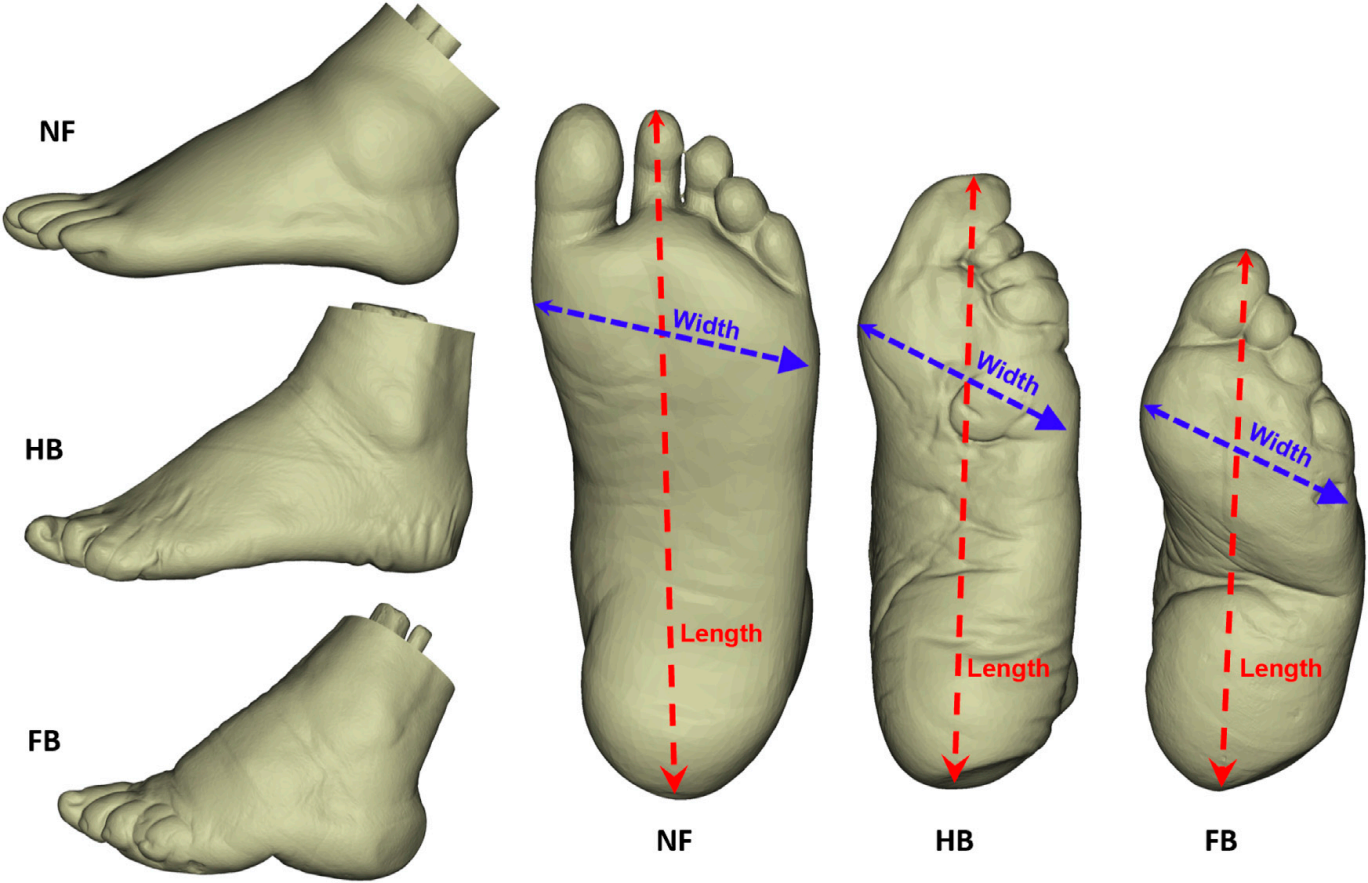
Lateral and plantar view of the NF, HB foot, and FB foot.

Three female participants joined this study, which was approved by the Ethical Committee in the Research Institute at Ningbo University (RAGH20170306). They were all elderly, and one exhibited a typical (normal) foot, one was classified as half-bound foot, and the final participant had a full-bound foot (see Figure 1 for foot shapes; Table 1 for all demographics). They were informed of the objectives, requirements, and procedures of this project and provided written consent.

**TABLE 1:** Demographics of participants.

	Normal foot	Half bound	Full bound
Age (yrs)	71	84	92
Height (m)	1.56	1.56	1.53
Mass (kg)	52.2	49.1	47.5
Foot length (mm)	214.06	202.81	165.86
Foot width (mm)	84.17	67.14	65.25

**FIGURE 2:**
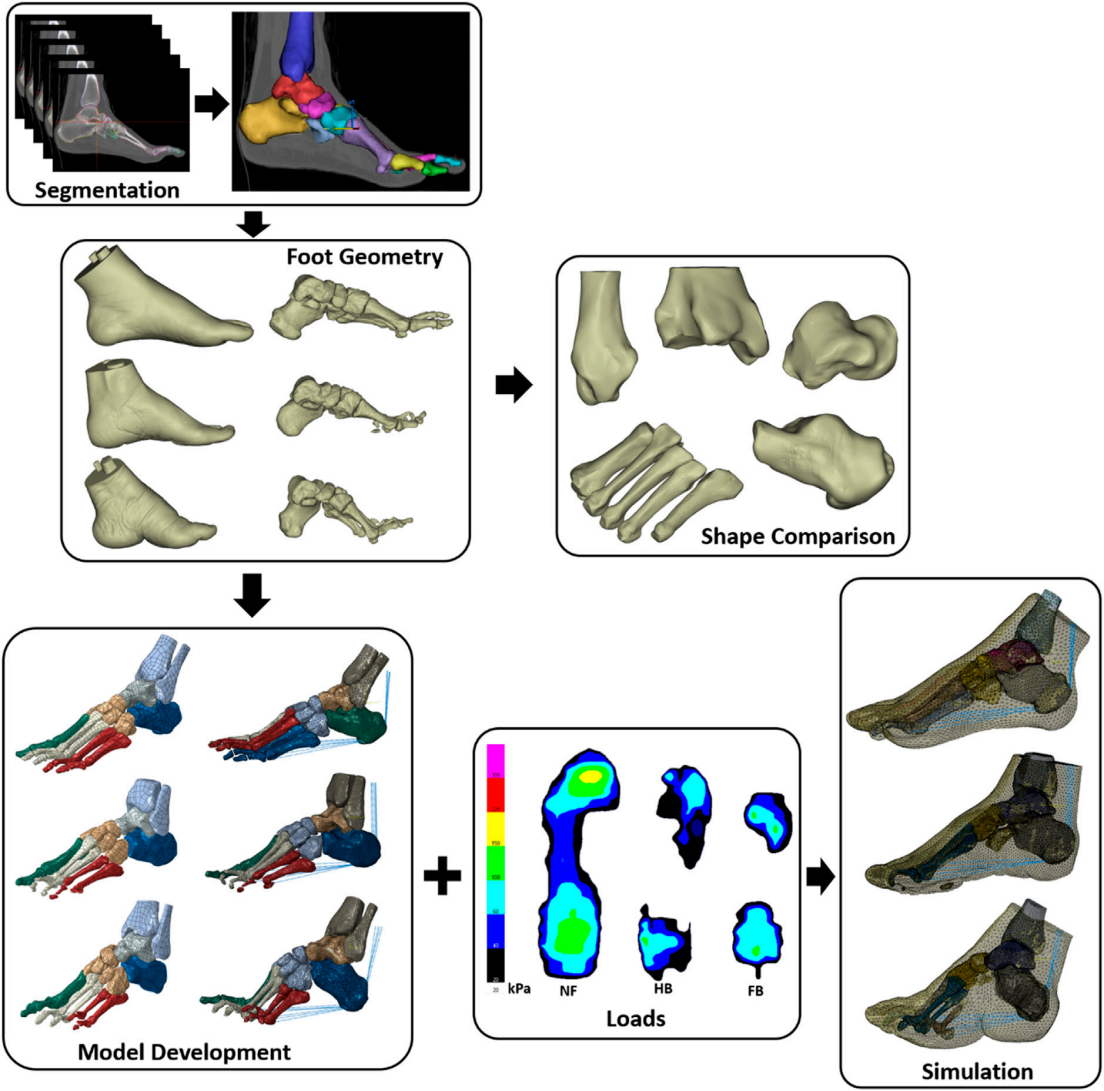
Framework of segmentation, shape comparison, FE model development, and simulation.

The complete framework for this study included segmentation of foot geometries for shape analysis and FE model construction for the NF, HB, and FB. We then integrated foot plantar pressure to simulate internal bone stress and predicted bone remodelling adaptation. Figure 2 highlights the steps and geometric models created for each stage of the computational pipeline.

### Collection and processing of experimental data

Participants attended the gait test and foot CT scanning sessions separately. The gait test session collected walking kinematics and plantar pressure of both feet, part of which were reported in our previous work (Gu et al., 2015; Zhang et al., 2015). Three participants walked with a Pedar pressure system (Novel Pedar System, Germany) to record the plantar pressure at a frequency of 50 Hz. Socks were worn to assist the fixation of the insole to the plantar surface. To represent the gait loading patterns during realistic scenarios, the walking velocity was not controlled. The plantar pressure from both feet was measured and averaged over three trials. The plantar pressure of the left foot from two consecutive steps was used in the current study.

The plantar surface was divided into separate regions including the hallux, other toes, medial forefoot, lateral forefoot, medial midfoot, lateral midfoot, medial rearfoot, and lateral rearfoot for the NF participant. The HB foot was divided into regions of hallux, medial forefoot, lateral forefoot, medial rearfoot, and lateral rearfoot, and the FB foot was divided into regions of medial forefoot, lateral forefoot, medial rearfoot, and lateral rearfoot. This was performed as the bound foot has distinctly different contact with the ground, and we aimed to contrast similar anatomical regions, which was published in our previous study (Gu et al., 2015).

We measured the discrete peak and mean plantar pressure values, the vertical ground reaction force, and the center of pressure (CoP) trajectory, following our previously established protocol (Mei et al., 2015; Mei et al., 2018). The CoP trajectory was categorised into the coordinate system of the ***x***-axis (medial–lateral) and ***y***-axis (anterior–posterior) as time-varying one-dimensional variables. The trajectories in the **x**-axis and **y**-axis were then normalised to the width and length of each subject-specific foot shape before performing time-series comparison (Pataky et al., 2014; Booth et al., 2018).

A total of six trials of left plantar pressure data were included in the comparative analysis, specifically the discrete values of mean and peak pressure in each anatomical region, and time-varying vertical ground reaction force and CoP trajectory in the medial–lateral (***x***-axis) and anterior–posterior (***y***-axis) directions. Gait plantar pressure distribution, CoP trajectory, and ground reaction forces are available to download from our open-source online repository (figshare: 10.17608/k6.auckland.19335488).

Following the gait test, participants attended a CT scanning session in the local hospital with the approved ethics. The CT scanning of the left and right feet was conducted using a SIEMENS Scanner (SIEMENS CT VA0 COAD, Munich, Germany) in a non-weight bearing and supine position. The parameters were set with a slice thickness of 0.6 mm and pixel size of 0.521 mm × 0.521 mm, with all CT images exported as a DICOM file for segmentation. The DICOM file of foot images was manually segmented in the Mimics 21.0 (Materialise, Leuven, Belgium) (Figure 2). Before exporting as separate geometrical (***stl***) parts, the bone and tissue geometries were first smoothed with a smooth factor of 0.4 and then wrapped to eliminate geometric gaps of less than 0.7 mm. The quality of each geometrical bone shape was checked using HyperMesh (2017, Altair, Troy, MI, United States of America) to ensure that the Jacobian of each element was greater than 0.3.

For bone shape comparison, the surface mesh (***.stl***) of the calcaneus, talus, tibia, fibula, tarsus (navicular, cuboid, and medial/intermediate/lateral cuneiform), and one to five metatarsals bones was organised into a vector matrix. The bone shapes are available to download from our open-source online repository (figshare: 10.17608/k6.auckland.19335395). Following a previously established framework of statistical shape modelling (SSM) (Zhang et al., 2014) using the musculoskeletal modelling software Gias2 (https://pypi.org/project/gias2/) developed at the Auckland Bioengineering Institute, a comparison of the shape of the HB, FB, and NF was made. Specifically, mesh fitting was first performed to ensure that the HB, FB, and NF had a consistent mesh topology using the iterative closest point and partial least squares fitting (Besl and McKay, 1992; Sarkalkan et al., 2014; Zhang et al., 2016). Second, the centroid alignment was performed for the three specific foot types (Zhang et al., 2014; Zhang et al., 2016; Mei et al., 2021; Xiang et al., 2022) for the last step of principal component analysis to compare the shape variations in the FB and HB against the NF bones. Finally, differences in bone shape were then plotted using error maps with open-source software CloudCompare (http://www.danielgm.net/cc/).

### Development of the computational finite element foot model

The foot bones and skin mesh were exported from Mimics as ***stl*** files. The geometries were post-processed in Geomagic Studio 12 (Research Triangle Park, NC, United States) to diagnose ill-conditioned elements and repair and then exported as surface files (***iges***) for meshing in HyperMesh 2017 (Altair Engineering Inc., Hyperworks, United States). Solid tetrahedral elements were then generated with an element size of 2.0 mm for bones and 3.0 mm for soft tissue. All meshes were quality checked before exporting as ***inp*** files for model assembly in Abaqus (Dassault Systems Simulia Corp., Johnston, United States). These geometry sizes also provided mesh convergence for the von Mises (VM) stress we were computing.

The bony structures were defined as linearly elastic isotropic materials with Young’s modulus of 7300 MPa and Poisson’s ratio of 0.3 (Cheung and Zhang, 2005), uniform density was assigned in both the cortical and trabecular bones, and the lumped soft tissue encapsulating the bone was assigned with a linear elastic model (Young’s modulus of 0.15 MPa and Poisson’s ratio of 0.45) (Cheung and Zhang, 2005). The tibia and fibula proximal surface were fixed as boundary conditions for a quasi-static simulation, and the plantar pressure was applied to the plantar surface of the foot mesh. Five linearly elastic connectors with a stiffness of 200N/mm were created to link the calcaneus notch with the base of the proximal phalanges (Kitaoka et al., 1994; Chen et al., 2019) to represent the plantar fascia. Five sets of rigid connector forces with a value assigned to 0.5 BW were applied to the Achilles tendon representing calf muscles during gait (Cheung et al., 2004). The foot FE models are available to download from our open-source online repository (figshare: 10.17608/k6.auckland.19335542).

The bone remodelling algorithm used is this study was adapted from the work of Doblaré and García (2002). The macroscopic apparent bone density is predicted based on the principles of continuum damage mechanics. This model has been shown to give anatomically and biologically realistic bone adaptation predictions in the proximal femur. Readers are referred to the work of Doblaré and García (2002), but the basic implementation in our Abaqus model is presented in Supplementary Appendix SA.

## Results

### Gait performance

**FIGURE 3:**
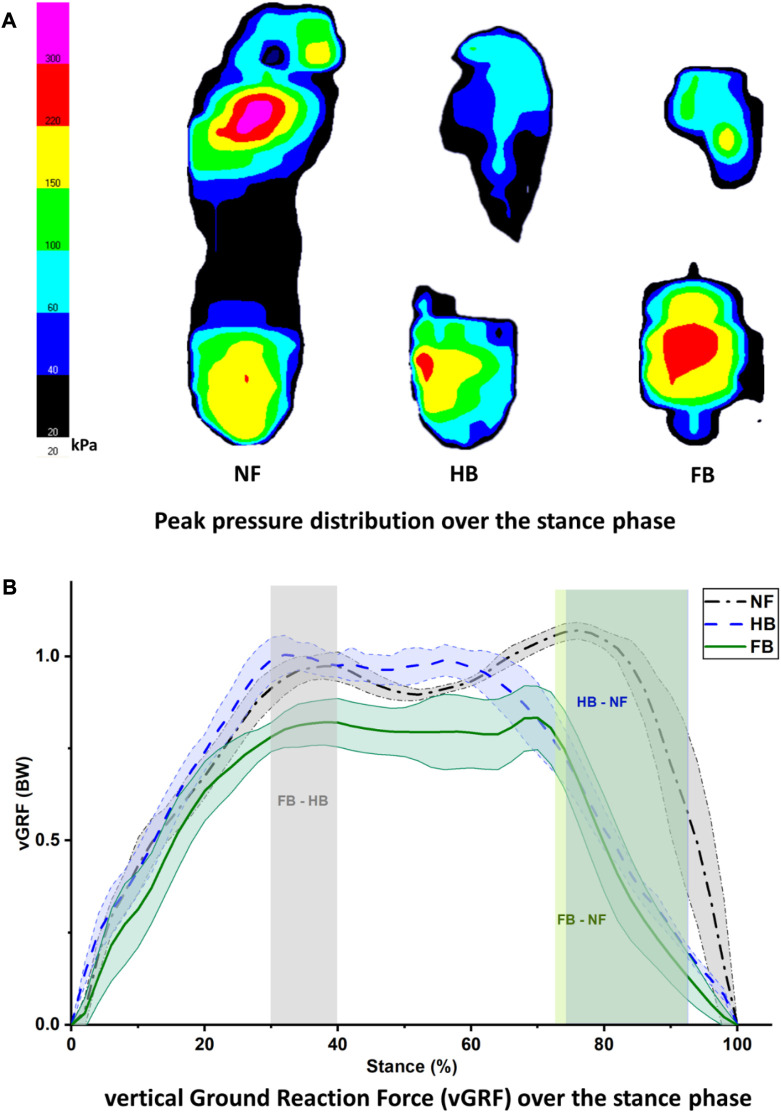
Peak pressure distribution during stance **(A)** and vertical ground reaction force **(B)** in the NF, HB, and FB foot types.

**FIGURE 4:**
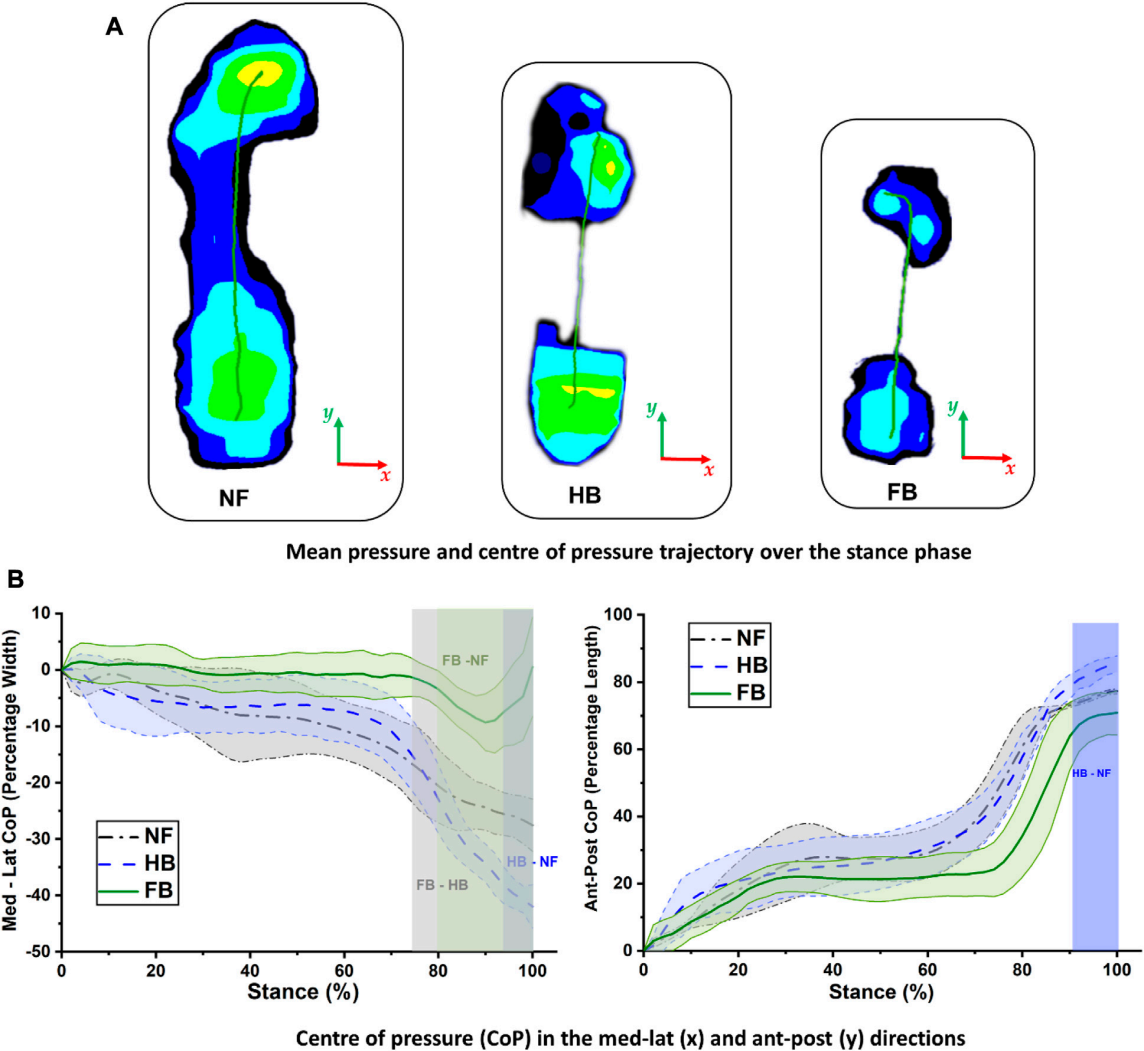
Center of pressure trajectory in the NF, HB foot, and FB foot during stance **(A)** and comparison of medial–lateral (left) and anterior–posterior (right) CoP trajectories **(B)**.

Figure 3A depicts the peak pressure distribution in the NF, HB foot, and FB foot. As illustrated in Figure 4A, the overall distribution of mean plantar pressure and CoP trajectory during stance is presented. Specifically, in the other toes (toes except hallux), the NF has larger pressure while no pressure is exhibited in HB and FB feet. Similarly for the midfoot, the NF presents larger pressure while no pressure is observed in HB and FB feet. However, the FB showed higher peak pressure than both the NF and HB. We used the mean pressure in each plantar region during stance as the boundary condition for the FE foot model. This is because the peak pressure typically resembled a small anatomical region and/or small time point during gait, which is less representative of the whole gait cycle.

Figure 3B presents the vertical ground reaction force during stance for all three feet. The NF participant presented a typical pattern of the vertical ground reaction force with the first peak of weight acceptance and the second peak of push-off. The difference in HB and FB differed significantly during the 30%–40% of the stance phase. The differences between HB and NF and FB and NF were observed at the second peak of push-off, specifically with an atypical pushing off during the 72%–92% (FB vs. NF) and 74%–92% (HB vs. NF) phases of stance.

The CoP trajectory was explored for the NF, HB foot, and FB foot. As shown in Figure 4A, during gait, the CoP trajectory starts at the heel and migrates towards the medial forefoot for both the NF and HB, whereas the CoP migrates towards the lateral forefoot for the FB. The medio-lateral CoP pathway (as a percentage of foot width) and anterior–posterior CoP pathway (as a percentage of foot length) were further explored to better understand the foot balance. Figure 4B **(left)** shows the medio-lateral CoP trajectory, and differences among the NF, HB foot, and FB foot were observed in late stance, during the push-off phase, with the greatest variation in the forefoot region. Specifically, the differences were observed in the 94%–100% phase of stance (HB and NF), 80%–100% phase of stance (FB and NF), and 76%–100% phase of stance (FB and HB). Figure 4B **(right)** shows the anterior–posterior CoP trajectory differences among the NF, HB foot, and FB foot were also observed in late stance. Specifically, the differences were observed in the 92%–100% phase of stance (HB and NF), 76%–84% phase of stance (FB and NF), and 94%–100% phase of stance (FB and HB).

### Geometric and shape differences

The foot profiles (length and width) of the three elderly females are shown in the present study (Figure 1). Specifically, the measured lengths and widths were 214.06 mm and 84.17 mm for the NF, 202.81 mm and 67.14 mm for the HB foot, and 165.86 mm and 65.25 mm for the FB foot, respectively.

Hence, as expected, the HB and FB feet exhibited shorter lengths compared to NF. Moreover, the FB exhibited a high arch in the midfoot forming an extreme dome, compared to the HB and NF. The HB also showed a higher arch compared to the NF.

**FIGURE 5:**
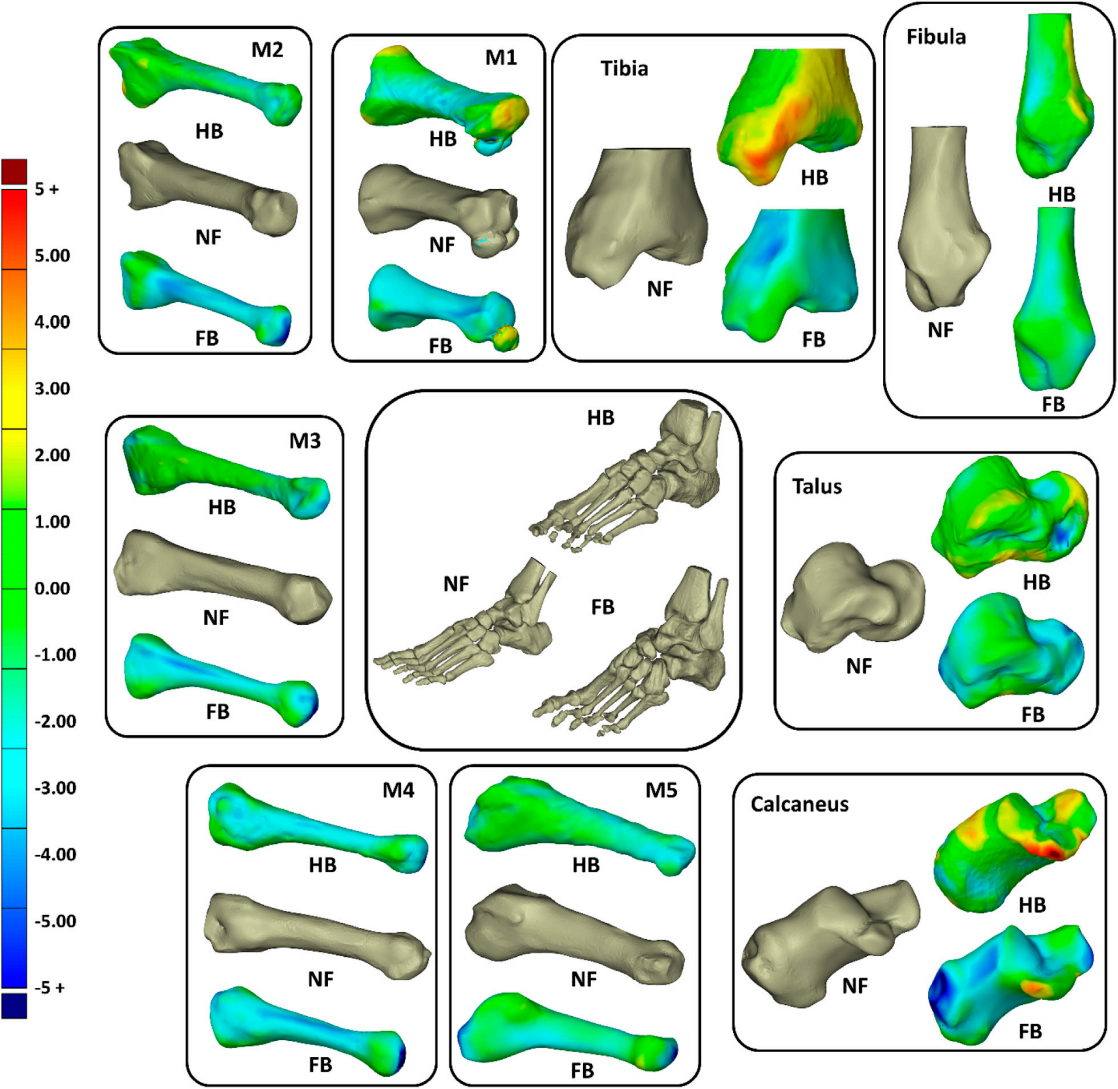
Overall shape comparisons of the calcaneus, talus, tibia, fibular, and metatarsal (M1–M5) bones of the NF, HB foot, and FB foot (consistent color scale with unit in mm).

To further elucidate the 3D shape differences in the foot bones, we morphed the FB and HB geometric bones (tibia, fibula calcaneus, talus, and one to five metatarsals) onto the NF as a reference. Figure 5 shows that, in general, the HB and FB feet had smaller metatarsals than the NF, but the HB was larger than FB. The HB and FB fibula was also similar in size but smaller than the NF. Full details of the shapes are presented in Supplementary Appendix SB *,* Supplementary Figure SB1 (calcaneus), Supplementary Figure SB2 (talus), Supplementary Figure SB3 (tibia), Supplementary Figure SB4 (fibula), Supplementary Figure SB5 (M1), Supplementary Figure SB6 (M2), Supplementary Figure SB7 (M3), Supplementary Figure SB8 (M4), and Supplementary Figure SB9 (M5), with quantification of Huasdorff distance and Gaussian distribution of the distances.

**FIGURE 6:**
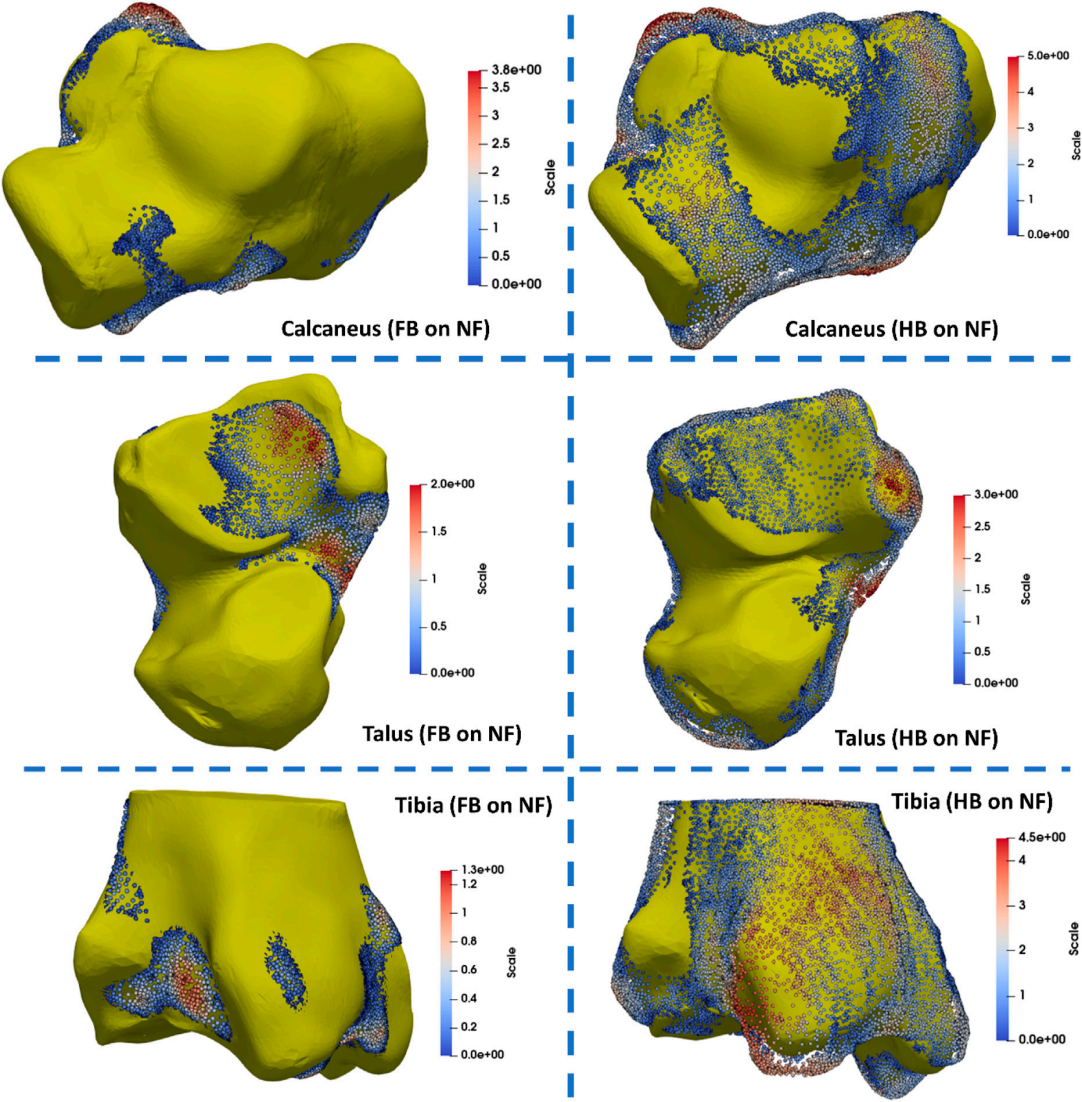
Quantification of regional shape variations in the calcaneus, talus, and tibia bones via mapping the FB and HB feet to the NF (unit in mm).

The tibia, talus, and calcaneus were the main bones presenting differences. The HB tibia, talus, and calcaneus presented larger features by 3–5 mm than the NF, whereas the FB was smaller than the NF by 3–5 mm. Specifically, the larger bone growth in the HB was observed primarily at the tibia–talus and talus–calcaneus articulations. As shown in Figure 6, the regional shape variations in vertices of calcaneus, talus, and tibia bones (hindfoot region) were quantified for illustration of the middle talar articular surface (calcaneus), posterior calcaneal articular surface (talus), and distal articular surface (tibia).

### Finite element simulations and CT validation

**FIGURE 7:**
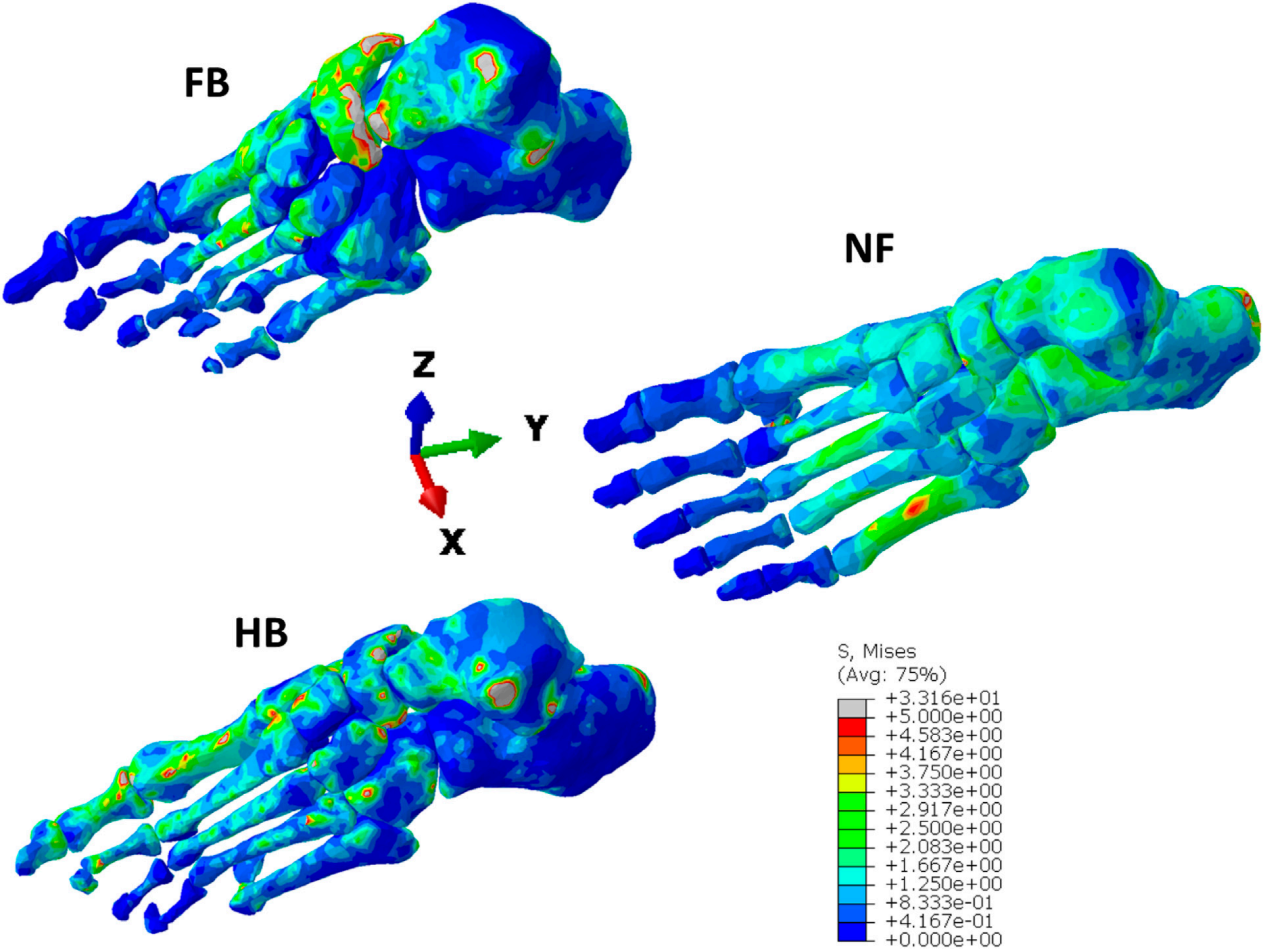
von Mises stress distribution in the NF, HB, and FB foot models.

Figure 7 presents the VM bone stress distributions in the NF, HB, and FB models from the finite element simulations of the stance phase of gait. The stress ranges from a low 0.4 MPa in non-directly loaded regions, up to 5 MPa in highly stressed regions. There are a few minor regions that experience loads up to 30 MPa near bone articulations. It is observed that the NF has a more uniform stress distribution up to the metatarsals (with a slight peak on M5) compared to the HB and FB. The HB shows greater peak VM stress in the first metatarsal and hallux, with peak VM stress evident in the midfoot and talus. In contrast, the FB has highly concentrated VM stress in the midfoot, with peak stresses in the calcaneus. Importantly, both the HB and FB do not show a uniform stress in the calcaneus compared with the NF.

**FIGURE 8:**
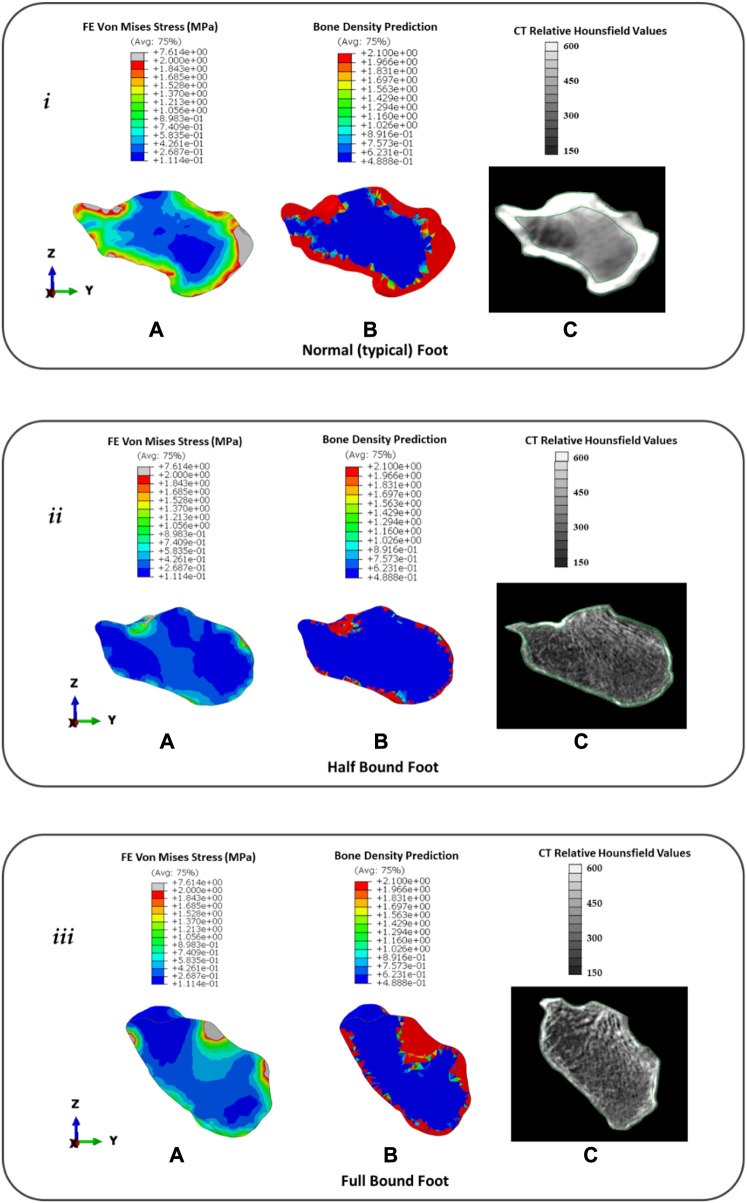
Distribution of FE predicted von Mises stress **(A)**, predicted bone density (g/cm^3^) using remodelling algorithm **(B)**, and CT with relative Hounsfield units **(C)** for the calcaneus in the typical (normal) NF (***i***), HB foot (***ii***), and FB foot (***iii***).

Figure 8 presents the (**a**) FE predicted VM stress for stance, (**b**) predicted bone remodelling adaptation, and (**c**) CT slice with relative Hounsfield units for the calcaneus in the NF (***i***), HB (***ii***), and FB (***iii***) feet. It should be noted that white regions represent the cortical bone in the CT image and gray regions represent the lumped trabecular bone. Further remodelling steps of the foot bones are presented in Supplementary Appendix SC.

In the NF (Figure 8), the FE model predicted a consistent intensity of increased VM stress in the outer cortical region with peaks (∼7.6 MPa) at the posterior Achilles tendon insert and anterior region articulating with the navicular bone. However, there was a low-stress region observed near the superior surface articulating with the talus. This was consistent with 60 days of predicted bone remodelling, showing increased thickening of the bone density in the regions with higher VM stress. A comparison with the CT slice of the same specimen showed CT evidence of the cortical bone uniformly around the outer edge with a thicker bone at the Achilles tendon insert. However, at the talus–calcaneus articulation, there is also CT evidence of the cortical bone in contrast to the FE model prediction.

In the HB (Figure 8), the FE model predicted a sparse VM stress in the outer cortical region with peaks (∼2 MPa) at the posterior Achilles tendon insert and superior region articulating with the talus bone. The magnitude and intensity of the VM stress are significantly lower than those of the NF. This was consistent with 60 days of predicted bone remodelling, showing increased thickening of the bone density primarily at the talus articulation. A comparison with the CT slice of the same specimen showed CT evidence of a thin cortical bone layer with increased density at the Achilles tendon inserts and talus articulation.

In the FB (Figure 8), the FE model predicted a sparse VM stress in the outer cortical region with peaks (∼7.6 MPa) at the posterior Achilles tendon insert and superior region articulating with the talus bone. The magnitude of the peaks was consistent with the NF but similar in pattern to the HB with peaks at bony articulation and tendon inserts. This was consistent with 60 days of predicted bone remodelling, showing increased thickening of the bone density primarily at the Achilles tendon insert and talus articulation. A comparison with the CT slice of the same specimen showed CT evidence of a thin cortical bone layer with an increased density at the Achilles tendon insert and an increased density of trabecular structures at the talus articulation.

## Discussion

Our models showed bound feet were shorter than typical feet due to bounding constraints, and this was consistent with other studies. For example, Qin et al. (2015) reported average lengths of 223 mm in bound feet and 254 mm for typical feet, and Reischl et al. (2008) reported a length of 18 cm for bound foot and 22 cm for typical feet. This shorter length is associated with compressed bones and a higher arch in the bound foot. Specifically, the extreme dome-like arch in the FB formed due to the vertically re-oriented calcaneus and metatarsals following lifelong foot binding constraints. However, the HB presented only deformed toes since the binding was released earlier in age giving the foot bones an opportunity to grow with a small foot arch. Our model findings were consistent with previous studies that analysed the bone re-alignment in bound feet. Specifically, an increased horizontal metatarsal angle in comparison to the NF (Ma et al., 2013) increased bending and rotational articulation between the calcaneus and metatarsals in the BF (Stone, 2012) and vertical orientation of BF calcaneus (Munk and Poon, 1996; Richardson, 2009; Howard and Pillinger, 2010; Gu et al., 2013).

Our statistical mapping of the FB and HB bones onto the NF revealed size and shape differences. We found that, in general, the bones were smaller in the forefoot (especially the metatarsals), and this was consistent with the lower mean and peak plantar pressure that the HB and FB feet experience due to binding. Recent paleopathology studies (Berger et al., 2019; Lee, 2019; Zhao et al., 2020) found a similar finding by comparing recovered bound foot remains to the bones in the normal foot. Macroscopic examination of the shapes reported that the metatarsals (particularly 2–5) reduced in length and presented thin, gracile shafts and small distal heads. Interestingly, they found that M1 was not always smaller, and this is consistent with our finding for M1, which showed increased FE stress relative to the other metartarsals and was also slightly larger at the ends, especially in the HB foot. The shape differences in the rearfoot exhibited more adaption due to form and function of loading absorption. In particular, we found that the articulating joint surfaces in the HB and FB had greater variation in size than the NF. Although there are few studies analysing bone shape in the bound foot, we did identify one study that showed the talus of bound feet has extended and flattened articular joint surfaces (Berger et al., 2019; Lee, 2019), consistent with our work. Furthermore, the calcaneal sulcus in the HB and FB was larger than that in the NF, and the likely functional reason for this alteration is the articulation with the talus must be a more stable joint as the rearfoot bears most loading during walking in the bound foot (HB and FB). Moreover, the increased size of the tibia and talus articulation supports this idea. It is as if the function of the tibia as a vertical load bearing beam is extended down to the calcaneus.

The toe plantar pressure is relatively small in the HB and NF, and there is no midfoot loading due to compromised structural support. However, the plantar pressure pattern is more focussed towards the rear with increased bound constraints. For example, in our model, the NF is fairly even in pressure between the rearfoot, midfoot, forefoot, and toes. However, the HB foot is focussed on the rearfoot and reduced on the forefoot and only the hallux, whereas the FB is solely focussed on the rearfoot and forefoot. This focus towards the rear of the foot is consistent with the idea that the rearfoot is an extension of the lower extremity for shock dampening in the Chinese bound foot (Reischl et al., 2008; Gu et al., 2015; Reznikov et al., 2017). Furthermore, the reduced mean plantar pressure at the rear in the HB and FB is consistent with the reduced ground reaction forces we found during the push-off phase of gait. This is consistent with the deformity in the forefoot and toes affecting the natural ankle rollover motion during gait, providing a limited ankle range of motion in the sagittal plane during stance (Gu et al., 2015).

We did not observe major differences in the CoP for the bound foot participants versus the NF except during the push-off phase. The CoP path from rear to hallux was consistent with normal walking (Reischl et al., 2008) although slightly more lateral, which is consistent with a recent report that CoP trajectory shifts laterally in old age (Sole et al., 2017). The primary difference was observed in the FB, where the CoP moved more laterally during push-off, which was not observed in the HB as the hallux was still articulating in the HB foot. It was also observed in the FB that the medial–lateral CoP only varied within 10% of the foot width. This suggests that the FB had limited supination–pronation motion, which was not observed in the HB foot. Furthermore, the anterior–posterior CoP of the FB foot occupied only 20% of the foot length for 70% of stance unlike the HB and NF. These two CoP patterns suggest that the rearfoot took most of the loading in the FB foot and implies that the FB foot is functioning more like a rigid shock mechanism.

The calcaneus was investigated for its function as a posterior balance support and shock absorption mechanism. We observed in the NF that the VM stress and predicted bone density were consistent around the cortical shell and higher at the Achilles tendon insert and joint articulation. We found that this was consistent with the CT evidence of relative Hounsfield data from that same subject; however, our model did not predict density at one aspect of the talus articulation. We attribute this to the fact that our model was limited to only looking at one pose of the foot (built from CT), which was loaded with the mean plantar pressure from stance. We did not consider the dynamic articulation of the joint that would have provided a complete loading from gait and loaded the calcaneus fully from different orientations. However, we suggest from our results that a static simple model using mean pressure from gait reveals a good prediction of bone adaptation without the need to consider other tasks. Our prediction of the HB and FB feet improved our confidence in our approach as it predicted a thin bone density along the cortical shell and increased density only at the talus articulation and Achilles tendon insertion site. The CT evidence for both revealed a thinner bone density along the shell and increased density and/or trabecular density at the same anatomical region as the model. The increased VM stress and bone density, which moved progressively posterior, were consistent with the increasing vertical orientation of the calcaneus from the HB to the FB foot. This was also consistent with a recent study that found considerable loss of trabecular density in the calcaneus bones of bound feet (Zhao et al., 2020); however, the trabecular anisotropy in the calcaneus of bound foot typically remains unchanged (Reznikov et al., 2017). While we did not characterise the anisotropy in our study, we did note that the trabecular lines still remain consistent across all three models even though the density has been reduced. This suggests that the underlying loading patterns of principal stress are still similar. The altered gait loading profiles (highly concentrated in the rearfoot but less in the forefoot and toe regions) led to the adaptation of subtalar motion and calcaneus bone remodelling properties, which may provide implications for similar loading redistribution profiles related form and function adaptation in the modern foot disorders.

A few considerations should be taken into account while acknowledging the findings from the current study. First, we only included a single representative of NF, HB, and FB female participants. These elderly females with half- and full-bound characteristics are the last generation, as this custom has been banned over a century ago, making it difficult to increase the sample size. It was rare to find and obtain all the information for the current participants. Second, the CT imaging data were acquired from a non-weight bearing position, which might not be the real geometrical condition during gait or other activities. However, we employed subject-specific foot shape, boundary conditions, and external loadings all from the same subjects. Last, the material properties assigned were macroscopic models using simplified linear elastic material properties as it was not possible to obtain bone properties for each participant as the CT was not taken using a CT phantom. We were primarily interested in the relative pattern of bone density for model evaluation and plotted the relative Hounsfield instead.

## Conclusion

In summary, to our knowledge, this study is the first investigation of form and function in the Chinese bound foot combining gait, shape analysis, computational stress, and bone density prediction. This study revealed an insightful form and function finding by considering an atypical foot model. When the foot is bound, the foot arch increases and the calcaneus orients vertically. This causes the tibia, which typically acts as a load transfer beam and shock absorber, to extend its function all the way through the talus to the calcaneus. This is evident in the bound foot by (**i**) reduced CoP movement in the medial–lateral direction, which suggests reduced supination–pronation; (**ii**) increased density and stress in the talus–calcaneus articulation; and (**iii**) increased bone growth in the bound foot at articulation joints in the tibia, talus, and calcaneus.

## Data Availability

The bone shape, gait loading, and foot FE model data for the analysis presented in this study are available for download from our online repository (bone shape: 10.17608/k6.auckland.19335395; gait plantar loading: 10.17608/k6.auckland.19335488; and foot FE model: 10.17608/k6.auckland.19335542).
