# Supplementary material for: Understanding the form and function in Chinese bound foot from last-generation cases
Source: Front Physiol. 2023 Sep 19;14:1217276. doi: 10.3389/fphys.2023.1217276 (PMC10545958; doi:10.3389/fphys.2023.1217276)

Supplementary Materials for

Understanding the Form and Function in Chinese Bound Foot from Last-Generation Cases

*Qichang Mei[1,2,3], *Yaodong Gu[1,2,3], Julie Kim[3], Liangliang Xiang[1,2,3], Vickie Shim[3], *Justin Fernandez[1,2,3,4]

[1]Faculty of Sports Science, Ningbo University, Ningbo, 315211, China
[2]Research Academy of Grand Health, Ningbo University, Ningbo, 315211, China
[3]Auckland Bioengineering Institute, The University of Auckland, Auckland, 1010, New Zealand
[4]Department of Engineering Science, The University of Auckland, Auckland, 1010, New Zealand

* Correspondence:
Address: Faculty of Sports Science, Ningbo University, No. 818, Fenghua Rd, Jiangbei District, Ningbo, Zhejiang, 315211, China
Dr. Qichang Mei, PhD (Email: qmei907@aucklanduni.ac.nz; meiqichang@outlook.com)
Prof. Yaodong Gu, PhD (Email: guyaodong@hotmail.com); A/Prof. Justin Fernandez, PhD (Email: j.fernandez@auckland.ac.nz)

Appendix A

Given the strain $\boldsymbol{\varepsilon}_t$, apparent density ρ_t and fabric tensor $\widehat{\boldsymbol{H}}_t$ for the current time step t, a mechanical stimulus $\boldsymbol{Y}_t$ is calculated.

$$\boldsymbol{Y}_t = 2\left[2\hat{G}\ \mathrm{sym}[(\boldsymbol{H}_t\boldsymbol{\varepsilon}_t\boldsymbol{H}_t)(\boldsymbol{H}_t\boldsymbol{\varepsilon}_t)] + \hat{\lambda}\ \mathrm{tr}\left({\boldsymbol{H}_t}^2\boldsymbol{\varepsilon}_t\right)\mathrm{sym}[\boldsymbol{H}_t\boldsymbol{\varepsilon}_t]\right], \quad (1)$$

where $\hat{G}$, and $\hat{\lambda}$ are the Lamé constants of ideal compact bone with null porosity, whose density is represented by $\hat{\rho}$, and $\boldsymbol{H}_t$ is a remodelling tensor given by.

$$\boldsymbol{H} = \left(\frac{\rho}{\hat{\rho}}\right)^{\beta(\rho)/4} A(\rho)^{1/4}\widehat{\boldsymbol{H}}^{1/2}. \quad (2)$$

A weighted sum of stimulus $\boldsymbol{J}_t$ between the spherical and deviatory part is calculated next

$$\boldsymbol{J}_t = (1-\omega)\ \mathrm{tr}(\boldsymbol{Y}_t)\tfrac{1}{3}\boldsymbol{1} + \omega\ \mathrm{dev}(\boldsymbol{Y}_t) = (1-2\omega)\ \mathrm{tr}(\boldsymbol{Y}_t)\tfrac{1}{3}\boldsymbol{1} + \omega\boldsymbol{Y}_t. \quad (3)$$

Using the scalar weighting factor of $\omega\ \in [0,\ 1]$.

Accounting for the weighted sum of stimulus $\boldsymbol{J}_t$ from the current loading configuration and the number of cycles of loading per day n_t, the resorption level g_t^r, and the apposition level g_t^f,are calculated

$$g_\mathrm{t}^r = \frac{\sqrt{2(1-\omega)}}{{n_t}^{1/m}\sqrt{B(\rho_t)}\hat{\rho}^{2-\beta(\rho_t)/8}A(\rho_t)^{1/8}27^{1/4}}\left({\boldsymbol{J}_t}^{-1}{:}{\boldsymbol{J}_t}^{-1}\right)^{1/4} - \frac{1}{(\psi_t^* - w){\rho_t}^{(16-5\beta(\rho_t))/8}}$$

$$g_\mathrm{t}^f = {n_t}^{1/m}\sqrt{B(\rho_t)}\hat{\rho}^{2-\beta(\rho_t)/8}A(\rho_t)^{1/8}\frac{3^{1/4}}{\sqrt{2(1-\omega)}}(\boldsymbol{J}_t{:}\boldsymbol{J}_t)^{1/4} - (\psi_t^* + w){\rho_t}^{(16-5\beta(\rho_t))/8}$$

Where ψ_t^* is the reference stimulus level, w is the half-width of the bone equilibrium zone, m is the empirical weighting between the importance of the load intensity and the number of load cycles to bone remodelling, and $B(\rho_t)$ and $A(\rho_t)$ are constants.

The resorption and apposition levels determine which remodelling criteria is active according to

$$
\begin{array}{rl}
\text{resorption if} & g_{\mathrm{t}}^{r} \geq 0 \text{ and } g_{\mathrm{t}}^{f} < 0 \\
\text{apposition if} & g_{\mathrm{t}}^{f} \geq 0 \\
\text{equilibrium if} & g_{\mathrm{t}}^{r} < 0 \text{ and } g_{\mathrm{t}}^{f} < 0
\end{array}
$$

Following this, the rates for surface remodelling $\dot{r}_{\mathrm{t}}$, density $\dot{\rho}_t$ and remodelling tensor $\dot{\boldsymbol{H}}_t$ are calculated

$$
\dot{r}_{\mathrm{t}} = \begin{cases} -c_r \dfrac{g_{\mathrm{t}}^{r}}{{\rho_t}^{2-\beta(\rho_t)/2}} & \text{resorption} \\ c_f \dfrac{g_{\mathrm{t}}^{f}}{{\rho_t}^{2-\beta(\rho_t)/2}} & \text{apposition} \end{cases}
$$

$$
\dot{\rho}_t = k \dot{r}_t {S_V}_t \hat{\rho}
$$

$$
\dot{\boldsymbol{H}}_t = \begin{cases} \dfrac{3\beta(\rho_t) k \dot{r}_{\mathrm{t}} {S_V}_t}{4\mathrm{tr}(\boldsymbol{H}_t^{-2} \boldsymbol{J}_t^{-3} \boldsymbol{H}_t : \widehat{\omega})} \dfrac{\hat{\rho}}{\rho_t} \boldsymbol{J}_t^{-3} : \widehat{\omega} & \text{resorption} \\ \dfrac{3\beta(\rho_t) k \dot{r}_{\mathrm{t}} {S_V}_t}{4\mathrm{tr}(\boldsymbol{H}_t^{-2} \boldsymbol{J}_t \boldsymbol{H}_t : \widehat{\omega})} \dfrac{\hat{\rho}}{\rho_t} \boldsymbol{J}_t : \widehat{\omega} & \text{apposition} \end{cases}
$$

Where c_r and c_f are the remodelling velocity for resorption and apposition, respectively, $S_V(n)$ is the specific surface (internal surface per unit volume), k is the ratio between the available surface for remodelling and the total internal surface, and $\widehat{\omega}$ is the fourth-order rank tensor form of ω. To avoid unphysiological values of density and numerical problems, the rates were adjusted to ensure that the density remains in the range of $0.01\,\mathrm{g/cm^3} \leq \rho \leq \hat{\rho}$ at all the time.

The variables are updated using the forward Euler method, and consequently the constitutive tensor is updated so that its local orthotropy directions coincides with those of the principal axes of the remodelling tensor, and stiffness in the material direction is a function of principal values of the remodelling tensor. This process repeats until the specified simulation period is reached. The parameter values used in the simulation are summarized in the ***Table A1*** below.

Table A1. Parameters used in the bone remodelling algorithm

Variable	Value
ω	0.3
ψ_t^*	50MPa/day
m	4
w	25% of ψ_t^*
c_r	0.02μm/day
c_f	0.02μm/da
k	100%

Appendix B

For bone shape comparison, the surface mesh (***.stl***) of the calcaneus, talus, tibia, fibula, tarsus (navicular, cuboid, medial/intermediate/lateral cuneiform), and 1-5 metatarsals bones were organized into a vector matrix. The bone shapes are available to download from our open-source online repository (figshare: 10.17608/k6.auckland.19335395).

As expected, the HB and FB feet exhibited shorter lengths due to the foot binding compared to the NF. Moreover, the FB exhibited a high arch in the midfoot forming an extreme dome, compared to the HB and NF. The HB also showed a higher arch compared to the NF. Full details of the bone shapes are presented below.

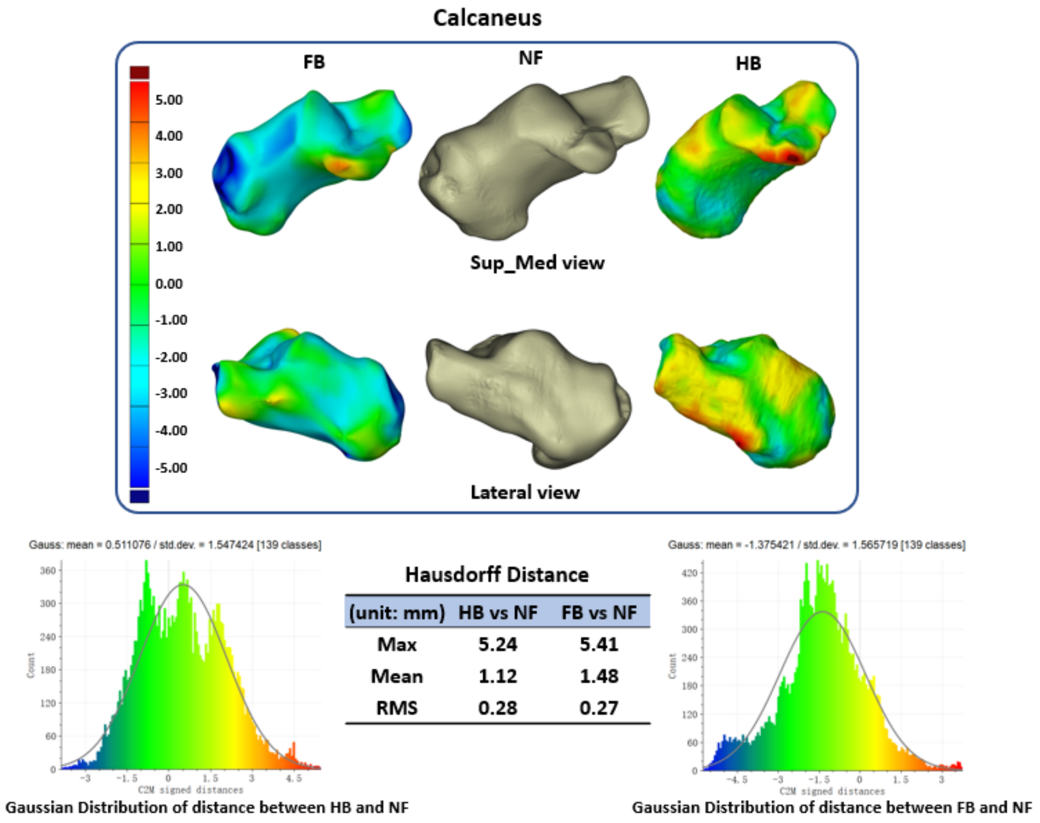

(unit: mm)	HB vs NF	FB vs NF
Max	5.24	5.41
Mean	1.12	1.48
RMS	0.28	0.27

Fig. B1. Illustration of shape differences in the calcaneus bone of HB and NF, and FB and NF with quantification of Hausdorff Distance, and Gaussian Distribution.

Talus

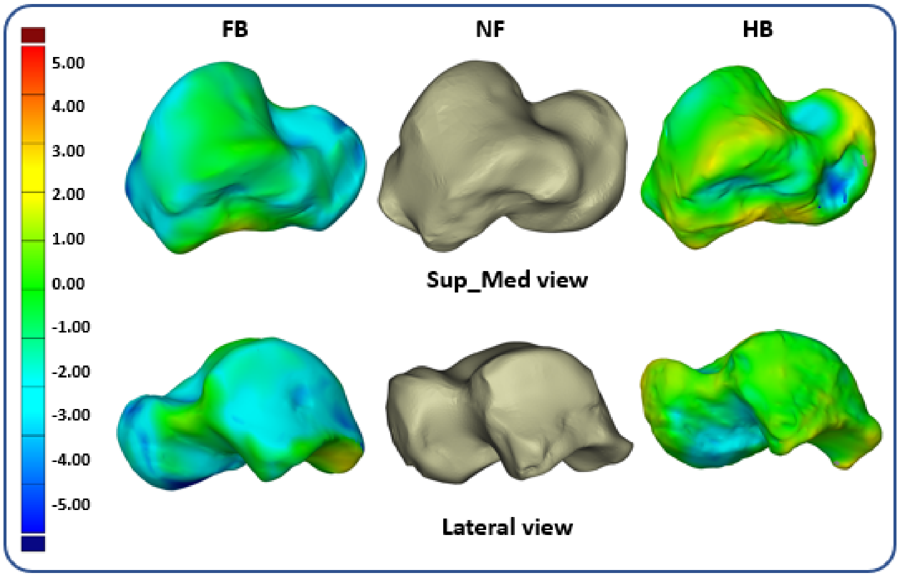

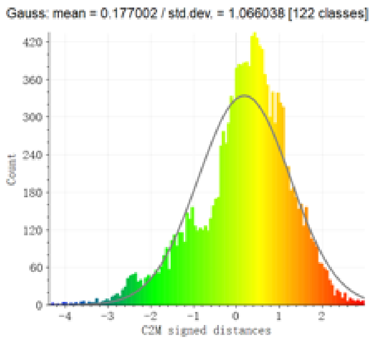

Gaussian Distribution of distance between HB and NF

Hausdorff Distance

(unit: mm)	HB vs NF	FB vs NF
Max	4.39	4.83
Mean	0.72	1.3
RMS	0.21	0.2

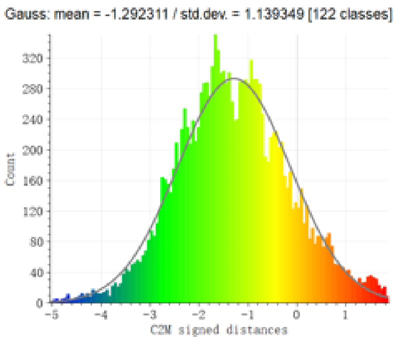

Gaussian Distribution of distance between FB and NF

Fig. B2. Illustration of shape differences in the talus bone of HB and NF, and FB and NF with quantification of Hausdorff Distance, and Gaussian Distribution.

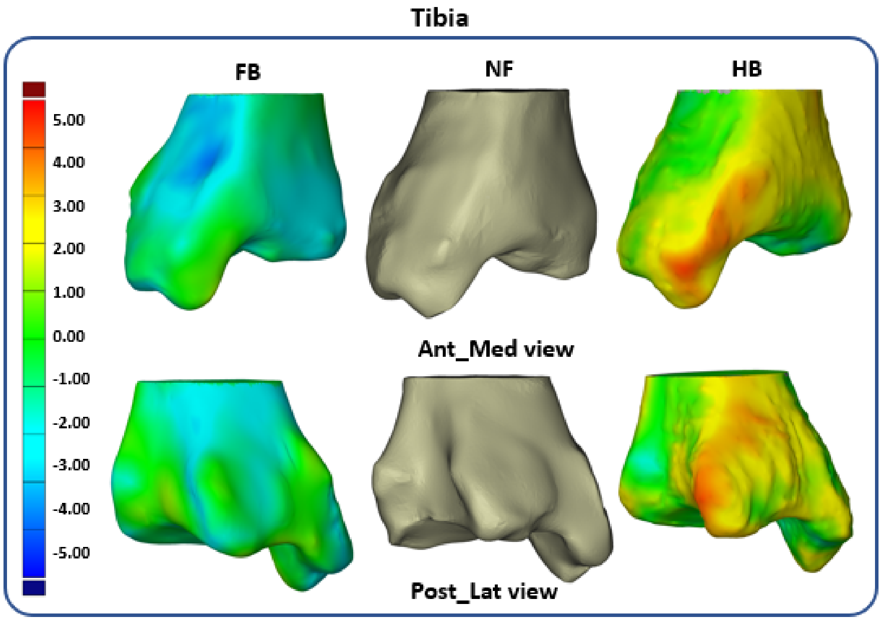

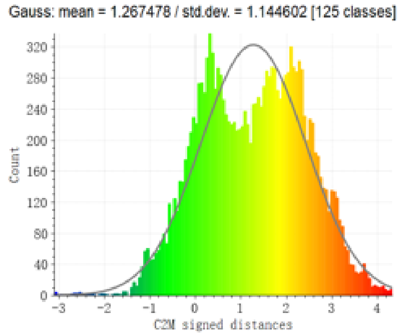

Gaussian Distribution of distance between HB and NF

Hausdorff Distance

(unit: mm)	HB vs NF	FB vs NF
Max	4.21	3.75
Mean	1.25	1.01
RMS	0.2	0.18

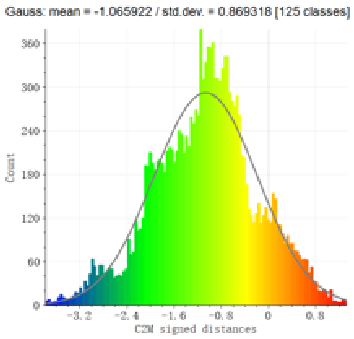

Gaussian Distribution of distance between FB and NF

Fig. B3. Illustration of shape differences in the tibia bone of HB and NF, and FB and NF with quantification of Hausdorff Distance, and Gaussian Distribution.

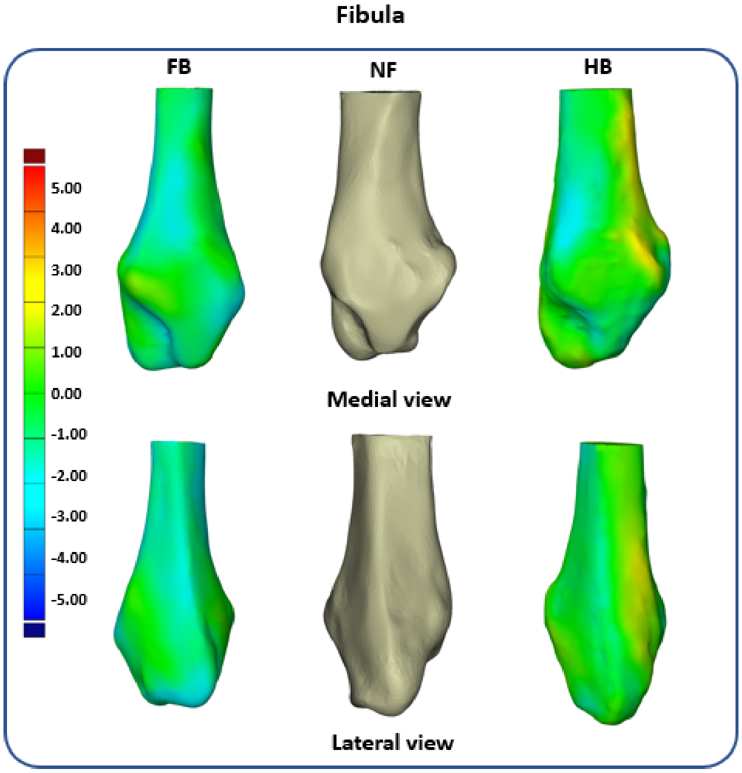

Hausdorff Distance

(unit: mm)	HB vs NF	FB vs NF
Max	2.28	3.21
Mean	0.56	0.8
RMS	0.19	0.19

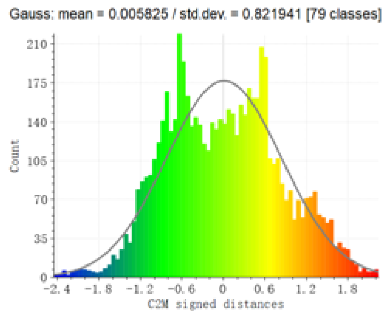

Gaussian Distribution of distance between HB and NF

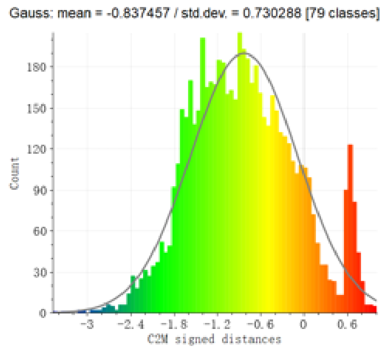

Gaussian Distribution of distance between FB and NF

Fig. B4. Illustration of shape differences in the fibula bone of HB and NF, and FB and NF with quantification of Hausdorff Distance, and Gaussian Distribution.

Hausdorff Distance

(unit: mm)	HB vs NF	FB vs NF
Max	4.34	6.79
Mean	0.92	1.86
RMS	0.24	0.22

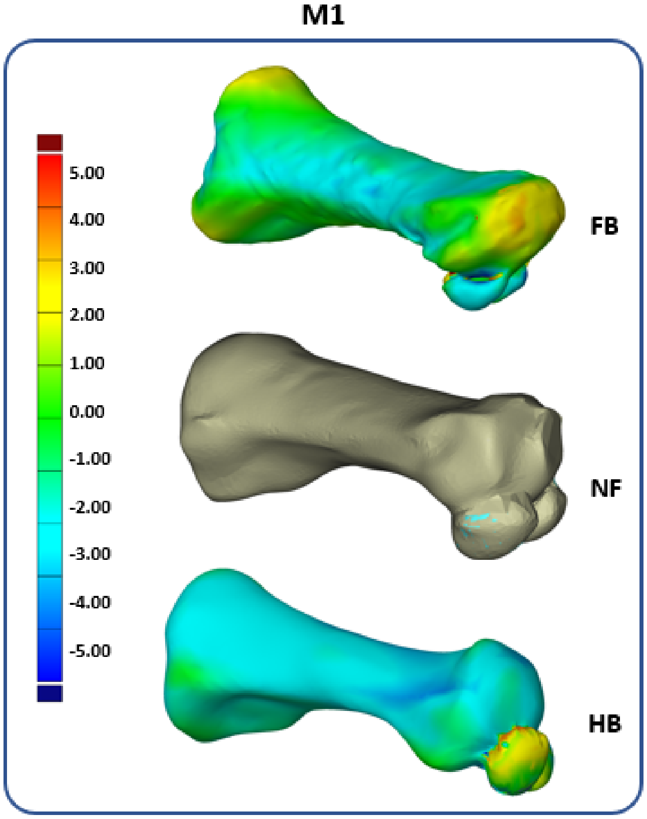

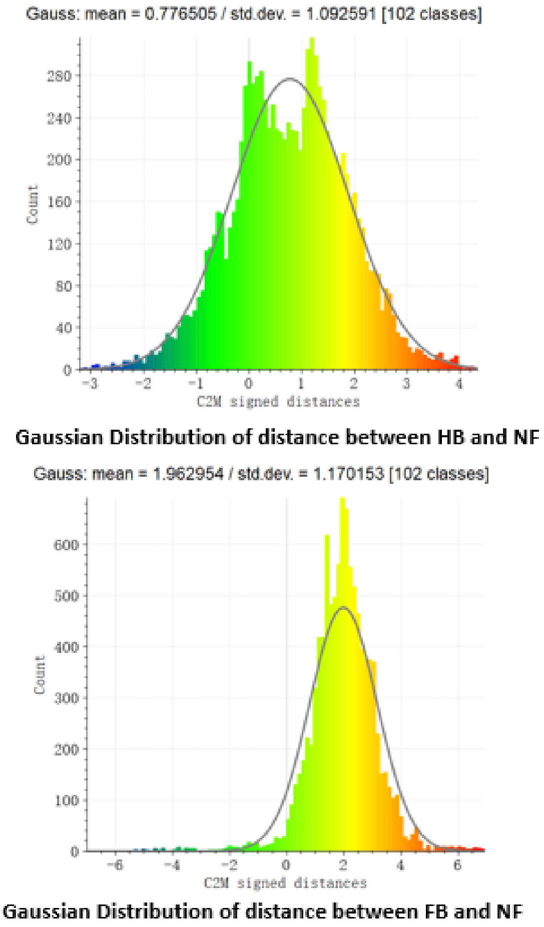

Fig. B5. Illustration of shape differences in the M1 bone of HB and NF, and FB and NF with quantification of Hausdorff Distance, and Gaussian Distribution.

M2

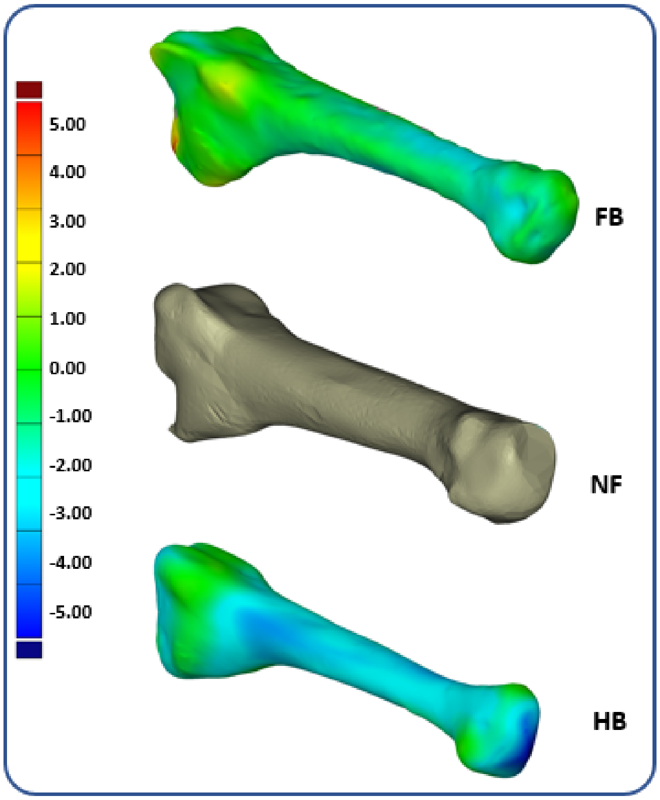

Hausdorff Distance

(unit: mm)	HB vs NF	FB vs NF
Max	2.78	7.14
Mean	0.71	1.66
RMS	0.27	0.27

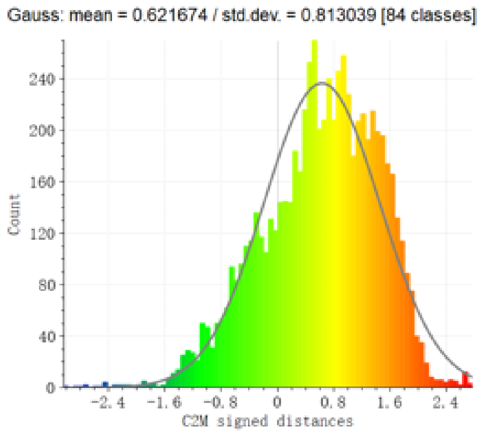

Gaussian Distribution of distance between HB and NF

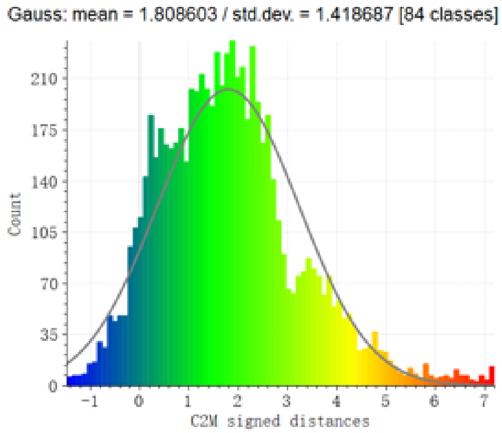

Gaussian Distribution of distance between FB and NF

Fig. B6. Illustration of shape differences in the M2 bone of HB and NF, and FB and NF with quantification of Hausdorff Distance, and Gaussian Distribution.

M3

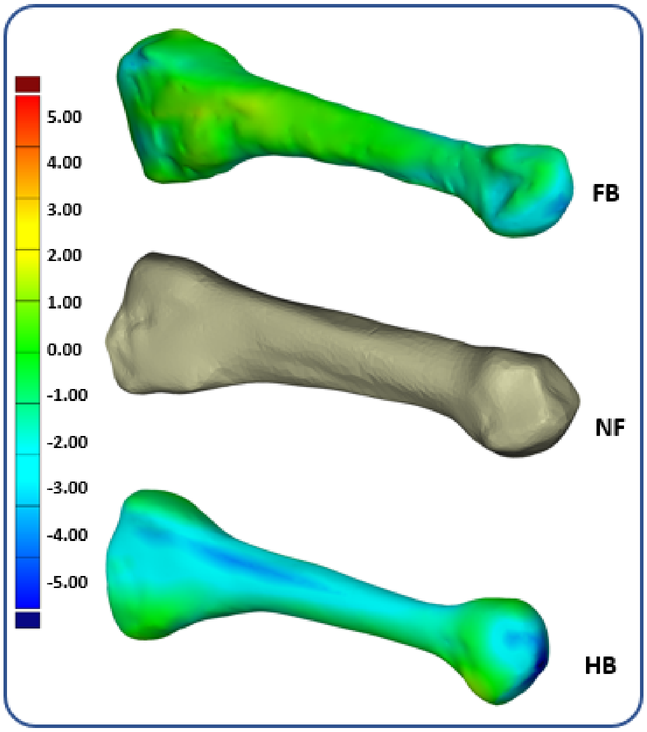

Hausdorff Distance

(unit: mm)	HB vs NF	FB vs NF
Max	3.72	6.26
Mean	0.94	1.68
RMS	0.26	0.26

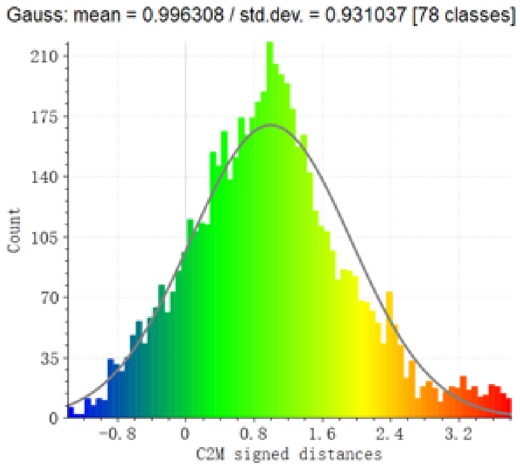

Gaussian Distribution of distance between HB and NF

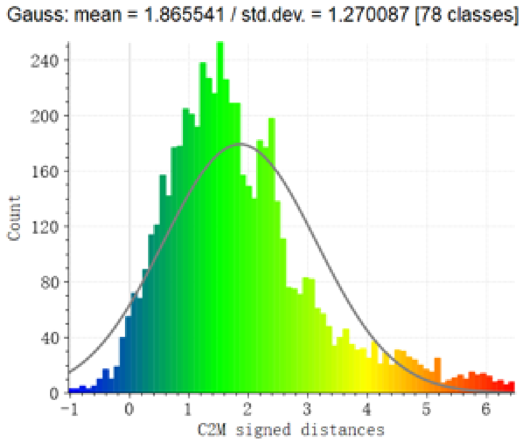

Gaussian Distribution of distance between FB and NF

Fig. B7. Illustration of shape differences in the M3 bone of HB and NF, and FB and NF with quantification of Hausdorff Distance, and Gaussian Distribution.

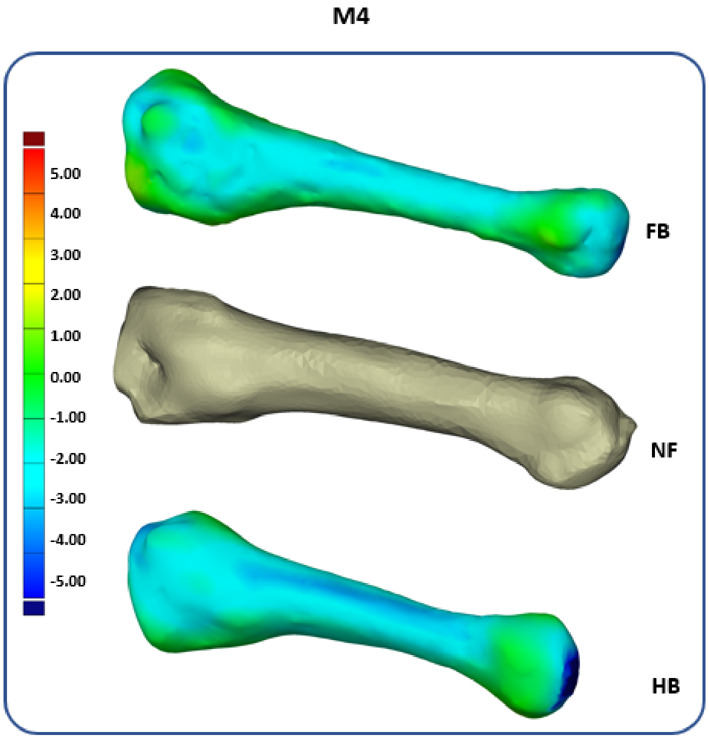

Hausdorff Distance

(unit: mm)	HB vs NF	FB vs NF
Max	4.66	10.34
Mean	1.39	3.03
RMS	0.26	0.25

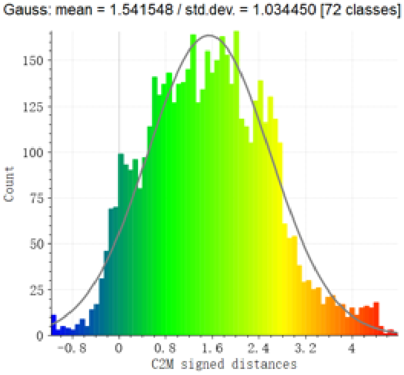

Gaussian Distribution of distance between HB and NF

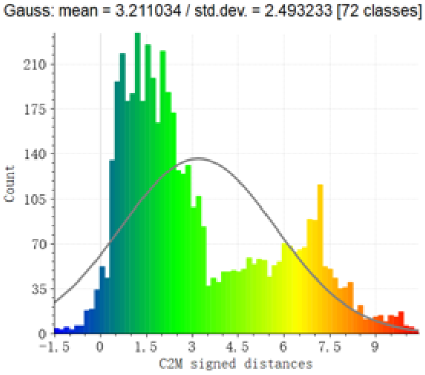

Gaussian Distribution of distance between FB and NF

Fig. B8. Illustration of shape differences in the M4 bone of HB and NF, and FB and NF with quantification of Hausdorff Distance, and Gaussian Distribution.

M5

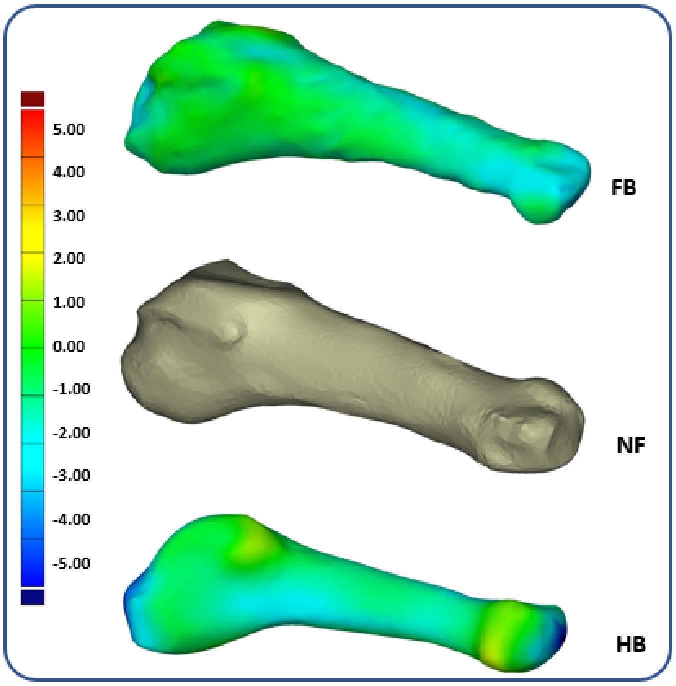

Hausdorff Distance

(unit: mm)	HB vs NF	FB vs NF
Max	3.72	6.82
Mean	1.04	2.3
RMS	0.25	0.25

Gauss: mean = 1.189630 / std.dev. = 0.783447 [72 classes]

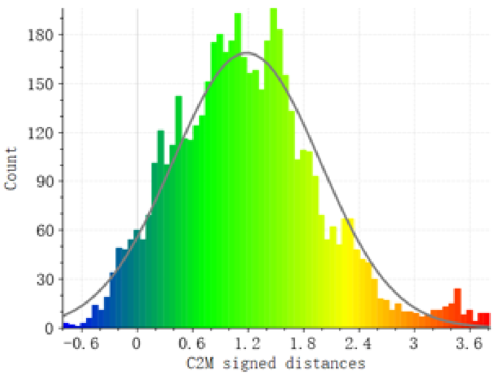

Gaussian Distribution of distance between HB and NF

Gauss: mean = 2.457517 / std.dev. = 2.007765 [72 classes]

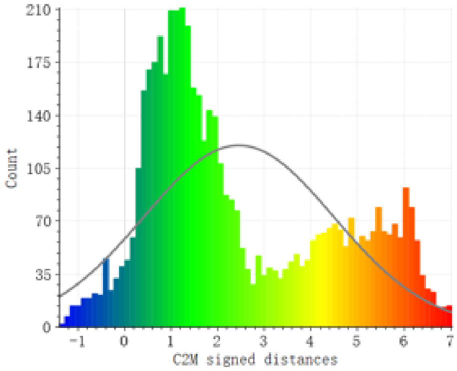

Gaussian Distribution of distance between FB and NF

Fig. B9. Illustration of shape differences in the M5 bone of HB and NF, and FB and NF with quantification of Hausdorff Distance, and Gaussian Distribution.

Appendix C

Appendix C presents the remodelling of foot bones in the FE model of normal foot (NF), half bound (HB) and full bound (FB).

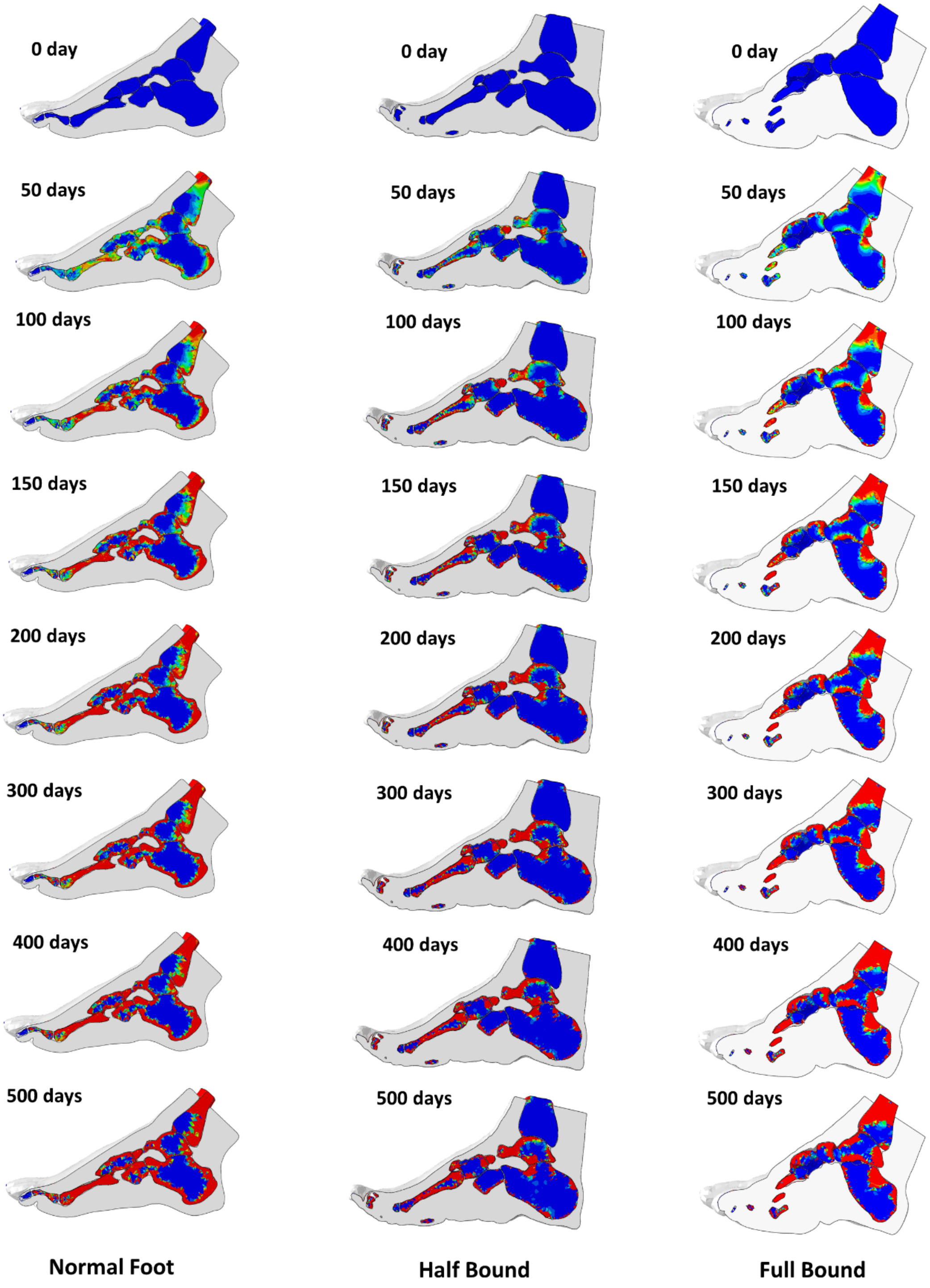

Supplement: Supplementary file 1 [file DataSheet1.pdf]
